# Gestational Diabetes, Subsequent Type 2 Diabetes, and Food Security Status: National Health and Nutrition Examination Survey, 2007–2018

**DOI:** 10.5888/pcd19.220052

**Published:** 2022-07-14

**Authors:** Lihua Li, Jiayi Ji, Yan Li, Yuanhui (Jasmine) Huang, Jee-Young Moon, Ryung S. Kim

**Affiliations:** 1 Department of Population Health Science and Policy, Icahn School of Medicine at Mount Sinai, New York, New York; 2 Institute for Healthcare Delivery Science, Icahn School of Medicine at Mount Sinai, New York, New York; 3 Tisch Cancer Institute, New York, New York; 4 Department of Biostatistics & Epidemiology, Rutgers School of Public Health, Piscataway, New Jersey; 5 Department of Obstetrics, Gynecology and Reproductive Science, Icahn School of Medicine at Mount Sinai, New York, New York; 6 Division of Hematology and Medical Oncology, Icahn School of Medicine at Mount Sinai, New York, New York; 7 Department of Epidemiology and Population Health, Albert Einstein College of Medicine, Bronx, New York

## Abstract

**Introduction:**

Despite many studies linking various risk factors to the association between gestational diabetes and subsequent type 2 diabetes, little is known about how food insecurity affects their association. We aimed to assess how the association between gestational diabetes and subsequent type 2 diabetes varies by food security status among women in the US.

**Methods:**

This study is a secondary data analysis of 9,505 US women aged 20 years or older who had at least 1 live birth; we used cross-sectional data from the National Health and Nutrition Examination Survey (NHANES) from 2007 through 2018. The main outcome was a diagnosis of type 2 diabetes in the subsequent years after the first live birth. We used multivariable survey-weighted negative binomial regressions to examine whether the association between gestational diabetes and subsequent type 2 diabetes differed by food security status, with and without adjusting for health behavior factors.

**Results:**

Gestational diabetes was significantly associated with subsequent type 2 diabetes (incidence rate ratio [IRR], 2.57; 95% CI, 2.45–2.69). The association between gestational diabetes and subsequent type 2 diabetes was significantly different by food security status (IRR, 2.34; 95% CI, 2.23–2.45 among food-secure women; IRR, 2.99; 95% CI, 2.70–3.28 among food-insecure women).

**Conclusion:**

The association between gestational diabetes and subsequent type 2 diabetes differs significantly by food security status. Public health and health care practitioners should consider food security status when designing and implementing diabetes prevention interventions for women with a history of gestational diabetes.

**Summary**

**What is already known on this topic?**

Gestational diabetes is strongly associated with subsequent type 2 diabetes.

**What is added by this report?**

The association between gestational diabetes and subsequent type 2 diabetes differs significantly by food security status.

**What are the implications for public health practice?**

Improving access to healthy food and reducing food insecurity may change the pathway between gestational diabetes and subsequent type 2 diabetes.

## Introduction

Gestational diabetes, defined as glucose intolerance with onset or first recognition during pregnancy, is one of the most common pregnancy complications (1,2). It affects up to 10% of pregnancies in the US (3). Among women with gestational diabetes, about 20% to 50% eventually develop type 2 diabetes (4,5). Previous studies confirmed that gestational diabetes is associated with both insulin resistance and impaired insulin secretion, and it shares the same risk factors with type 2 diabetes, such as family history, age, and body mass index (BMI) (6,7). Shortly after delivery, most women usually return to normal glucose regulation. However, women with a history of gestational diabetes have an increased risk of developing type 2 diabetes later in life compared with women without a history of gestational diabetes (6,8).

Food insecurity is defined as a lack of physical and economic access to sufficient, safe, nutritious food that meets the dietary needs of a person for an active and healthy life (9). Previous studies suggest that food insecurity may act as a risk factor for type 2 diabetes (10,11). Nevertheless, in the literature that links gestational diabetes to subsequent type 2 diabetes while accounting for demographic factors, socioeconomic status, lifestyles, and biomarkers (12–14), no study has examined the role of food security in the association between gestational diabetes and subsequent type 2 diabetes.

A healthy diet is an important factor for preventing the development and further complications of diabetes (15,16). However, the impact of food security on diabetes and its consequences have not been fully explored, especially among women with a history of gestational diabetes. The knowledge gap may be due to several reasons, such as data availability, varying definitions of food security, and its inconsistent measurement.

In this study, we aimed to address this research gap by examining whether and how much food security affects the association between gestational diabetes and subsequent type 2 diabetes. We hypothesized that the relative risk of developing subsequent type 2 diabetes — comparing women with a history of gestational diabetes to their counterparts without a history of gestational diabetes — in the food-insecure population is higher than in the food-secure population.

## Methods

We used publicly available cross-sectional data from the National Health and Nutrition Examination Survey (NHANES) from 2007 through 2018. Of all NHANES surveys, the 2017–2018 cycle of NHANES had the most up-to-date data on food security at the time our study was conducted (17). NHANES is a national survey conducted in the civilian, noninstitutionalized US population with a stratified, multistage, probability sampling design (18). The survey examines a nationally representative sample of approximately 5,000 persons each year and assesses their health and nutritional status. It has a unique feature of combining home-based interviews for demographic, socioeconomic, dietary, and health-related information, with physical examinations for medical, dental, and physiological measurements, as well as laboratory tests (18). This study involved secondary analysis of nationally representative survey data, which are publicly available and do not contain personal identifiers; therefore, the study was exempt from institutional review.

### Study sample

We first extracted data on 10,504 women aged 20 years or older who had at least 1 live birth. After excluding 120 (1.2%) survey participants with unknown food security status, we excluded 175 (1.8%) participants who had diabetes or borderline diabetes before pregnancy by checking if their age at diagnosis was younger than their age at first live birth (Figure). We then excluded 243 (2.4%) participants who had borderline gestational diabetes, which was defined as having been told by a doctor or other health professional that she had borderline diabetes during pregnancy. Borderline diabetes is a condition in which blood glucose is high, but not high enough to be diabetes (fasting plasma glucose of 100–125 mg/dL and 2-hour postprandial glucose of 140–199 mg/dL) (19). In addition, we excluded 461 (4.1%) participants who had missing values on key covariates, including 20 participants with missing values for age at diagnosis of type 2 diabetes. Our final study sample included 9,505 participants.

**Figure:**
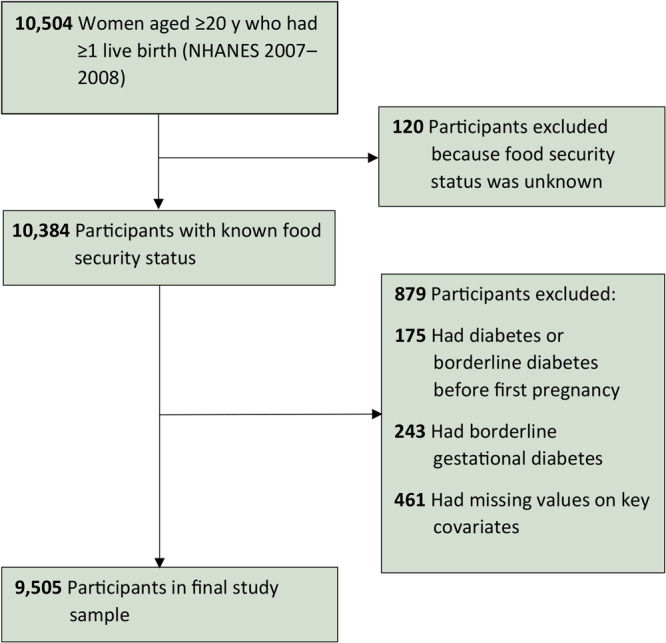
Analytic sample from 2007–2018 National Health and Nutrition Examination Survey. Covariates were age at first live birth, education, race and ethnicity, nativity, parity, family history of diabetes, and diabetes-related health behavior variables measured at the time of interview: alcohol use, total sugar intake 24 hours before the interview, smoking status, having rigorous-intensity activity at work or at leisure, body mass index, health insurance, and having a routine place for health care.

### Exposure variable

The exposure variable was gestational diabetes, which was categorized as a binary variable (yes/no). Participants who answered yes to the question “During pregnancy, were you ever told by a doctor or other health professional that you had diabetes, sugar diabetes or gestational diabetes? Please do not include diabetes that you may have known about before the pregnancy” were defined as having had gestational diabetes.

### Outcome variable

The outcome of interest was the development of type 2 diabetes after pregnancy, defined as a woman having ever been told by a doctor or health professional that she has diabetes or sugar diabetes. The participant with a history of gestational diabetes was considered to have subsequent type 2 diabetes if she had ever been told by a doctor or health professional that she had diabetes or sugar diabetes, and the age at diagnosis of diabetes was greater than the age at diagnosis of gestational diabetes. A participant without gestational diabetes was considered to have type 2 diabetes if she had ever been told by a doctor or health professional that she had diabetes or sugar diabetes, and the age at diagnosis of type 2 diabetes was greater than the age of her first live birth.

### Length of time at risk of subsequent type 2 diabetes

The duration of follow-up since first pregnancy was considered the length of time at risk of subsequent type 2 diabetes. For women who developed type 2 diabetes, it was calculated according to the participant’s age at first live birth, which was derived from their response to the question “How old were you at the time of your first live birth?” and age at diagnosis of type 2 diabetes, which was derived from their response to the question “How old were you when a doctor or other health professional first told you that you had diabetes or sugar diabetes?” We then calculated the difference in years between age at first live birth and age at diagnosis of type 2 diabetes. For women who did not develop type 2 diabetes, we calculated length of time at risk as the difference in years between age at first live birth and age at the NHANES interview.

### Food security variable

Food security was determined by examining answers to the questions from the US Food Security Survey Module, which is a well-validated questionnaire developed by the US Department of Agriculture; the module assesses household food security during the 12 months before the NHANES interview (20). It includes 18 items, 10 of which refer to adults in the household and 8 of which refer to children younger than 18 years. Because the study sample included adults only, we used the 4-level adult food security variable to classify participants into 2 groups: we considered participants with full food security and marginal food security to be food secure, and low food security and very low food security to be food insecure (20).

### Covariates

We included the following demographic and socioeconomic factors: age at first live birth, education (less than high school diploma, high school graduate, some college, and college graduate or higher), race and ethnicity (non-Hispanic White, non-Hispanic Black, Hispanic, and “other” [Mexican American, other Hispanic, and multiracial]), nativity (US born and non–US born), and parity (1 or 2, 3, and ≥4 children).

We also included family history of diabetes (yes/no) and diabetes-related health behavior variables measured at the time of interview: alcohol use (yes/no), total sugar intake 24 hours before the interview (mg), smoking status (current, ever, never), having rigorous-intensity activity at work or at leisure (yes/no), and BMI (normal weight, BMI <25.0; overweight, BMI 25.0–29.9; obese, BMI ≥30.0). In addition, we included health insurance (yes/no) and having a routine place for health care (yes/no).

### Statistical analysis

We performed descriptive analyses to summarize characteristics of women with and without gestational diabetes by food security status. To compare the difference between women who had gestational diabetes and women who did not, we used survey design–based *t* tests for continuous variables and Rao–Scott χ^2^ tests for categorical variables.

Considering each woman’s length of time at risk of subsequent type 2 diabetes, we first used a multivariable survey-weighted negative binomial regression (21) to examine the association between gestational diabetes incidence and subsequent type 2 diabetes incidence, adjusting for all the aforementioned covariates, and tested whether this association differed by food security status by testing the interaction of gestational diabetes and food security status. We further conducted subgroup analyses to examine this association stratified by food insecurity and food security. In both overall and subgroup analyses, the regression modeled the incidence rate of subsequent type 2 diabetes with the log of follow-up time since the first pregnancy as an offset and adjusted for covariates including and excluding health behavior factors.

We performed all analyses by using R package *survey* in R version 4.0.2 (R Core Team). Multiple imputation was not considered because the proportion of survey participants with missing values was low (<5%). All tests were 2-sided, with *P* < .05 considered significant. All analyses accounted for the complex survey design, including survey strata, clusters, and weights (22). We tabulated the incidence rate of subsequent type 2 diabetes, the ratio of incidence rates, and their 95% CIs. All percentages were survey weighted to be generalizable to the noninstitutionalized population of women in the US (23).

## Results

Of the 9,505 women, 7,326 (77.3%) were food secure, among whom 597 (8.0%) had gestational diabetes. Correspondingly, 2,179 (22.7%) women were food insecure, among whom 212 (9.7%) had gestational diabetes. Among women who were food secure, those who had gestational diabetes were more likely than those who did not have gestational diabetes to be younger at the time of interview (mean age, 52 [SD, 7.2] y vs 56 [SD, 2.5] y; *P* < .001) and at the time of the subsequent type 2 diabetes diagnosis (mean age, 41.7 [SD, 5.1] y vs 54.6 [SD, 3.2] y; *P* < .001), to consume more sugar (mean, 99.2 [SD, 27.0] mg vs 98.0 [SD, 18.0] mg; *P* = .76), be obese (56.4% vs 38.5%; *P* < .001), and have a family history of diabetes (64.5% vs 39.0%; *P* < .001) (Table 1). Among women who were food insecure, the pattern was similar except for health insurance and health behavior: women who had gestational diabetes were more likely than those who did not have gestational diabetes to have insurance (77.2% vs 73.0%; *P* = .65) and have vigorous activity both at work (21.0% vs 19.0%; *P* = .62) and during leisure time (12.3% vs 9.7%; *P* = .16).

**Table 1:** Characteristics of Survey Participants Included in Analysis (N = 9,505), by Food Security Status, National Health and Nutrition Examination Survey, 2007–2018^a^

Characteristic	Food security			Food insecurity		
	Gestational diabetes (n = 597)	No gestational diabetes (n = 6,729)	*P* value^b^	Gestational diabetes (n = 212)	No gestational diabetes (n = 1,967)	*P* value^b^
Age at first live birth, mean (SD), y	24 (1.5)	23 (0.9)	<.001	22 (2.5)	21 (0.9)	<.001
Age at interview, mean (SD), y	52 (7.2)	56 (2.5)	<.001	49 (12.1)	52 (5.2)	<.001
Follow-up time, median (IQR), y	28 (23–33)	33 (27–39)	<.001	27 (19–35)	31 (23–39)	<.001
**Race and ethnicity**						
Black, non-Hispanic	103 (7.9)	1,356 (10.2)	.005	44 (15.5)	493 (18.5)	.25
Hispanic	169 (12.5)	1,663 (11.3)		78 (28.2)	770 (25.4)	
Other^c^	81 (9.3)	689 (6.6)		14 (7.2)	118 (6.7)	
White, non-Hispanic	244 (70.3)	3,021 (71.9)		76 (49.1)	586 (49.5)	
**Education**						
Less than high school diploma	133 (15.0)	1,684 (15.1)	.003	76 (30.3)	820 (34.0)	.11
High school diploma/GED	115 (16.2)	1,631 (25.2)		48 (25.4)	464 (25.8)	
Some college or associate degree	202 (32.2)	1,982 (32.0)		76 (35.2)	551 (31.6)	
College degree or above	147 (36.6)	1,432 (27.7)		12 (9.1)	132 (8.6)	
**US born**						
No	153 (15.2)	1,832 (15.1)	.43	68 (24.1)	667 (22.2)	.64
Yes	444 (84.8)	4,897 (84.9)		144 (75.9)	1,300 (77.8)	
**Parity^d^**						
1 or 2	288 (54.1)	3,144 (52.4)	.36	84 (46.1)	765 (45.3)	.37
3	172 (25.9)	1,862 (27.8)		53 (24.2)	576 (29.6)	
≥4	137 (20.0)	1,723 (19.8)		75 (29.7)	626 (25.1)	
**Health insurance**						
No	122 (14.1)	1,040 (11.4)	.002	56 (22.8)	554 (27.0)	.65
Yes	475 (85.9)	5,689 (88.6)		156 (77.2)	1,413 (73.0)	
**Routine place for health care**						
No	68 (10.4)	577 (6.9)	.02	39 (22.6)	298 (16.0)	.25
Yes	529 (89.6)	6,152 (93.1)		173 (77.4)	1,669 (84.0)	
**Family history of diabetes**						
No	199 (35.5)	3,869 (61.0)	<.001	78 (31.8)	935 (46.6)	.004
Yes	398 (64.5)	2,860 (39.0)		134 (68.2)	1,032 (53.4)	
**BMI**						
Normal weight (BMI <25.0)	125 (20.5)	1,941 (32.1)	<.001	28 (11.2)	376 (21.9)	.006
Overweight (BMI 25.0–29.9)	141 (23.1)	2,059 (29.4)		44 (15.4)	514 (25.8)	
Obese (BMI ≥30.0)	331 (56.4)	2,729 (38.5)		140 (73.5)	1,077 (52.4)	
**Having rigorous activity at work** e						
No	520 (88.3)	5,985 (86.7)	.19	172 (79.0)	1,636 (81.0)	.62
Yes	77 (11.7)	744 (13.3)		40 (21.0)	331 (19.0)	
**Having rigorous activity at leisure^f^**						
No	516 (81.9)	5,755 (81.3)	.59	186 (87.7)	1,789 (90.3)	.16
Yes	81 (18.1)	974 (18.7)		26 (12.3)	178 (9.7)	
**Alcohol use**						
No	169 (23.8)	2,525 (29.0)	<.001	75 (28.7)	730 (29.5)	.67
Yes	428 (76.2)	4,204 (71.0)		137 (71.3)	1,237 (70.5)	
**Tobacco use**						
No	212 (35.8)	2,057 (32.8)	<.001	77 (38.9)	572 (33.7)	.002
Former	253 (47.4)	3,318 (52.5)		104 (45.9)	1,104 (51.2)	
Current	132 (16.5)	1,354 (14.7)		31 (15.2)	291 (15.1)	
**24-h Sugar intake, mean (SD), mg**	99.2 (27.0)	98.0 (18.0)	.76	118.2 (27.2)	107.2 (27.3)	.48
**Subsequent type 2 diabetes**						
Yes	148 (21.9)	679 (9.7)	<.001	51 (27.6)	271 (12.8)	<.001
No	449 (78.1)	6,050 (90.3)		161 (72.4)	1,696 (87.2)	
**Age at subsequent diagnosis of type 2 diabetes, mean (SD), y**	41.7 (5.1)	54.6 (3.2)	<.001	36.3 (6.9)	50.4 (4.5)	<.001

Abbreviations: BMI, body mass index; GED, General Educational Development.

a All data are shown as number (percentage) unless otherwise noted.

b *P* values calculated by using survey design–based *t* tests for continuous variables and Rao–Scott χ^2^ tests for categorical variables.

c “Other” includes Mexican American, other Hispanic, and multiracial.

d Number of children.

e Having rigorous-intensity activity that causes large increases in breathing or heart rate for at least 10 minutes continuously at work.

f Having rigorous-intensity activity that causes large increases in breathing or heart rate for at least 10 minutes continuously at sports, fitness, or recreational activity.

In the overall population, both gestational diabetes and food security status were significantly associated with type 2 diabetes. The ratio of incidence rates of type 2 diabetes, comparing those who had gestational diabetes with those who did not, was 2.57 (95% CI, 2.45–2.69) while adjusting for all the covariates including health behavior variables (Table 2). The ratio of incidence rates of type 2 diabetes, comparing those who had food security to those who did not, was 0.66 (95% CI, 0.56–0.77). The association of gestational diabetes and subsequent type 2 diabetes differed significantly by food security status (*P* value for interaction = .03). The analyses excluding 6 health behavior variables yielded similar results (Table 2).

**Table 2:** Incidence Rate Ratio (IRR) of Type 2 Diabetes Subsequent to Pregnancy That Compares Women With Gestational Diabetes With Those Without Gestational Diabetes in the Overall Sample, National Health and Nutrition Examination Survey, 2007–2018

Main covariates	IRR (95% CI) [*P* value^a^]			
	With adjustment of health behavior variables^b^		Without adjustment of health behavior variables^c^	
	Model with main effect	Model with interaction term	Model with main effect	Model with interaction term
**Gestational diabetes**				
No	1 [Reference]	1 [Reference]	1 [Reference]	1 [Reference]
Yes	2.57 (2.45–2.69) [<.001]	2.34 (2.23–2.45) [<.001]	2.59 (2.51–2.67) [<.001]	2.38 (2.30–2.46) [<.001]
**Food security status**				
Food insecurity	1 [Reference]	1 [Reference]	1 [Reference]	1 [Reference]
Food security	0.66 (0.56–0.77) [.008]	0.62 (0.56–0.90) [.01]	0.68 (0.61–0.76) [<.001]	0.62 (0.47–0.76) [<.001]
**Food security × gestational diabetes**	—	0.82 (0.68–0.90) [.03]	—	0.81 (0.74–0.87) [.004]

Abbreviation: — , does not apply.

a Determined by likelihood ratio test; *P* < .05 considered significant.

b Adjusted for age at first live birth, education, race and ethnicity, nativity, parity (number of children), family history of diabetes, health insurance, having a routine place for health care, and health behavior variables, which include body mass index, having rigorous-intensity activity at work, having rigorous-intensity activity at leisure, 24-hour sugar intake, alcohol use, and tobacco use.

c Adjusted for age at first live birth, education, race and ethnicity, nativity, parity (number of children), family history of diabetes, health insurance, and having a routine place for health care.

Overall, the incidence rates of subsequent type 2 diabetes among women with and without gestational diabetes were 9.82 and 4.31 per 1,000 person-years (Table 3) with a median follow-up of 28 years (interquartile range [IQR], 24–32 y) and 33 years (IQR, 28–38 y), respectively. The incidence rates of subsequent type 2 diabetes among women with and without gestational diabetes were 9.91 and 4.38 per 1,000 person-years in the food-secure group, respectively, and 9.60 and 4.44 per 1,000 person-years in the food-insecure group, respectively. The adjusted incidence rate ratio of having subsequent type 2 diabetes (comparing women with gestational diabetes to those without gestational diabetes) was 2.34 (95% CI, 2.23–2.45, *P* < .001) in the food-secure group and 2.99 (95% CI, 2.70–3.28, *P* < .001) in the food-insecure group. The estimates were similar when we removed 6 health behavior variables. In the overall and subgroup analyses, we found that obesity, having no routine place for health care, and having a family history of diabetes were significantly associated with the incidence of subsequent type 2 diabetes (Table 4).

**Table 3:** Incidence Rate Ratio (IRR) of Type 2 Diabetes Subsequent to Pregnancy, by Food Security Status, National Health and Nutrition Examination Survey, 2007–2018

Food security status	Weighted %	Type 2 diabetes events/total (weighted %)	Weighted incidence rate per 1,000 person-years	With adjustment of health behavior variables^a^		Without adjustment of health behavior variables^b^	
				IRR (95% CI)	*P* for interaction of gestational diabetes and food security^c^	IRR (95% CI)	*P* for interaction of gestational diabetes and food security^c^
**Overall (N = 9,505)**							
No gestational diabetes	91.8	950/8,696 (10.4)	4.31	1 [Reference]	—	1 [Reference]	—
Gestational diabetes	8.2	199/809 (23.8)	9.82	2.57 (2.45–2.69)		2.58 (2.50–2.66)	
**Food security (n = 7,326)**							
No gestational diabetes	92.2	679/6,729 (9.7)	4.38	1 [Reference]	.03	1 [Reference]	.004
Gestational diabetes	7.8	148/597 (21.9)	9.91	2.34 (2.23–2.45)		2.38 (2.30–2.46)	
**Food insecurity (n = 2,179)**							
No gestational diabetes	91.0	271/1,967 (12.8)	4.44	1 [Reference]		1 [Reference]	
Gestational diabetes	9.0	51/212 (27.6)	9.60	2.99 (2.70–3.28)		2.98 (2.77–3.19)	

Abbreviation: —, not applicable.

a Adjusted for age at first live birth, education, race and ethnicity, nativity, parity (number of children), family history of diabetes, health insurance, having a routine place for health care and health behavior variables, which include body mass index, having rigorous-intensity activity at work, having rigorous-intensity activity at leisure, 24-hour sugar intake, alcohol use, and tobacco use.

b Adjusted for age at first live birth, education, race and ethnicity, nativity, parity (number of children), family history of diabetes, health insurance, and having a routine place for health care.

c Determined by likelihood ratio test; *P* < .05 considered significant.

**Table 4:** Incidence Rate Ratio (IRR) of Type 2 Diabetes Subsequent to Pregnancy That Compares Women With Gestational Diabetes With Those Without Gestational Diabetes, by Food Security Status, National Health and Nutrition Examination Survey, 2007–2018

Covariates	Food security		Food insecurity	
	IRR (95% CI)	*P* value^a^	IRR (95% CI)	*P* value^a^
**Gestational diabetes**				
No	1 [Reference]		1 [Reference]	
Yes	2.34 (2.23–2.45)	<.001	2.99 (2.70–3.28)	<.001
**BMI**				
Normal weight (BMI <25.0)	1 [Reference]		1 [Reference]	
Overweight (BMI 25.0–29.9)	1.94 (1.30–2.91)	.002	2.07 (1.05–2.98)	.03
Obese (BMI ≥30.0)	5.27 (3.37–8.29)	<.001	5.78 (1.39–9.50)	.01
**Having rigorous activity at work^b^**				
No	1 [Reference]		1 [Reference]	
Yes	0.82 (0.54–1.25)	.22	0.99 (0.63–1.55)	.61
**Having rigorous activity at leisure^c^**				
No	1 [Reference]		1 [Reference]	
Yes	0.67 (0.46–1.01)	.06	0.82 (0.71–1.15)	.18
**24-h Sugar intake, mg**	0.97 (0.90–1.04)	.32	0.96 (0.88–1.04)	.46
**Alcohol use**				
No	1 [Reference]		1 [Reference]	
Yes	0.76 (0.64–0.96)	.04	0.98 (0.81–1.19)	.11
**Tobacco use**				
Never	1 [Reference]		1 [Reference]	
Former	0.94 (0.75–1.16)	.27	1.11 (0.92–1.40)	.40
Current	1.03 (0.94–1.12)	.22	1.24 (0.99–1.36)	.32
**Parity^d^**				
1 or 2	1 [Reference]		1 [Reference]	
3	0.92 (0.75–1.17)	.52	0.98 (0.86–1.24)	.50
≥4	1.04 (0.84–1.33)	.66	1.21 (0.99–1.44)	.30
**Health insurance**				
No	1 [Reference]		1 [Reference]	
Yes	1.11 (0.83–1.57)	.50	1.30 (0.98–1.54)	.40
**Routine place for health care**				
No	1 [Reference]		1 [Reference]	
Yes	2.09 (1.31–3.40)	.003	2.52 (1.37–3.86)	.02
**US born**				
No	1 [Reference]		1 [Reference]	
Yes	1.10 (0.84–1.50)	.50	1.36 (0.98–1.62)	.56
**Race and ethnicity**				
Black, non-Hispanic	1.41 (1.12–1.79)	.01	1.78 (1.38–2.20)	.02
Hispanic	1.44 (1.09–1.90)	.01	1.55 (1.04–2.00)	.04
Other^e^	2.23 (1.48–3.40)	<.001	2.66 (1.74–3.67)	.01
White, non-Hispanic	1 [Reference]		1 [Reference]	
**Education**				
Less than high school diploma	1 [Reference]		1 [Reference]	
High school diploma/GED	0.51 (0.39–0.67)	<.001	0.70 (0.54–0.77)	.01
Some college or associate degree	0.72 (0.52–1.01)	.08	0.94 (0.76–1.12)	.15
College degree or above	0.45 (0.33–0.68)	<.001	0.67 (0.53–0.71)	.005
**Age at first live birth, y**	0.97 (0.96–0.98)	<.001	1.01 (1.00–1.03)	.04
**Family history of diabetes**				
No	1 [Reference]		1 [Reference]	
Yes	3.54 (2.88–4.44)	<.001	4.02 (2.86–5.11)	<.001

Abbreviations: BMI, body mass index; GED, General Educational Development.

a Determined by likelihood ratio test; *P* < .05 considered significant.

b Having rigorous-intensity activity that causes large increases in breathing or heart rate for at least 10 minutes continuously at work.

c Having rigorous-intensity activity that causes large increases in breathing or heart rate for at least 10 minutes continuously at sports, fitness, or recreational activity.

d Number of children.

e “Other” includes Mexican American, other Hispanic, and multiracial.

## Discussion

To our knowledge, our study is the first to assess how the association between gestational diabetes and subsequent type 2 diabetes varies by food security status among women in the US. We found that the incidence rate ratio of subsequent type 2 diabetes (gestational diabetes vs no gestational diabetes) among women who were food secure at the time of interview was about 20% lower (2.34 vs 2.99) than it was among women who were food insecure. This difference in the association of gestational diabetes and subsequent type 2 diabetes between the 2 food security groups was significant, regardless of adjustment for health behavior factors. These findings provide the first pieces of evidence that food insecurity may be an intervenable risk factor in the association between gestational diabetes and development of type 2 diabetes. Further studies are needed to examine whether the change in food security status can mitigate the development of type 2 diabetes.

Our findings imply that public health and health care practitioners need to consider food security status in designing and implementing diabetes prevention interventions (24). In addition, existing government programs such as the Supplemental Nutrition Assistance Program (SNAP) and the Special Supplemental Nutrition Program for Women, Infants, and Children (WIC) should be expanded to reduce food insecurity (25). More efforts should be made to support enrollment in SNAP and WIC among women living in communities that are socially and economically marginalized. These efforts may include funding community-based organizations that focus on food insecurity; increasing acceptance of SNAP and WIC at online food retailers; and partnering with local communities to gain trust and improve food systems. During the COVID-19 pandemic, when food insecurity increased, the need for expanding and improving SNAP and WIC was magnified (26,27).

Our results demonstrate an opportunity for federal, state, and local officials to reduce the incidence of type 2 diabetes through appropriately addressing the issue of food insecurity, especially among women with a history of gestational diabetes. A recent study found that people who were food insecure were responsible for an additional $77.5 billion in health care expenditures annually compared with those who were food secure (28). This evidence provides additional incentives for stakeholders to take immediate action against food insecurity because of the potential savings to the health care system.

### Limitations

This study has several limitations. First, gestational diabetes and other variables were self-reported and were not verified by medical records, and thus may be subject to recall and response biases. The counts of gestational diabetes and type 2 diabetes may be underestimated because of undiagnosed cases. However, a study showed reasonable agreement (ie, 93.8%) between self-reported gestational diabetes and verification from birth certificates (29). Second, because food security status may not necessarily stay the same from the pregnancy to the time of interview, we were not able to establish temporality or causality in the relationship between food security and the progression of type 2 diabetes from gestational diabetes. Because our assumption was that current food security status is a proxy for past status, our results should be interpreted with caution. The limitation of assuming food security status stays the same from pregnancy to the time of interview also calls for future studies, particularly large longitudinal studies, to validate our findings. Also, data on BMI and health behavior factors, such as work and leisure-time activity, were collected either during the survey or when physical examinations were done. Their values do not concomitantly reflect participants’ health behavior between pregnancy and subsequent type 2 diabetes, and reverse causality may occur (ie, instead of the health behavior contributing to subsequent type 2 diabetes, type 2 diabetes changes one’s reported health behavior). Therefore, we performed analyses with and without adjusting for these variables as a bias analysis strategy for validating our findings.

### Conclusion

Gestational diabetes is strongly associated with subsequent type 2 diabetes, and this association may vary by food security status. Our findings suggest that improving access to healthy food and reducing food insecurity may change the pathway between gestational diabetes and subsequent type 2 diabetes. Interventions tailored to food-insecure women with a history of gestational diabetes are warranted.
